# Epigenetic Mechanisms in Penile Carcinoma

**DOI:** 10.3390/ijms140610791

**Published:** 2013-05-23

**Authors:** Hellen Kuasne, Fabio Albuquerque Marchi, Silvia Regina Rogatto, Ilce Mara de Syllos Cólus

**Affiliations:** 1 Department of General Biology, Londrina State University, Londrina, PR 86055-900, Brazil; E-Mails: hellenkuasne@hotmail.com (H.K.); colus@sercomtel.com (I.M.S.C.); 2 International Research and Teaching Center, CIPE, AC Camargo Cancer Center, São Paulo, SP 01508-010, Brazil; 3 Inter-institutional Grad Program on Bioinformatics, Institute of Mathematics and Statistics, USP—São Paulo University, São Paulo, SP 05508-090, Brazil; E-Mail: fmarchibr@hotmail.com; 4 Department of Urology, Faculty of Medicine, UNESP, Botucatu, SP 18618-970, Brazil

**Keywords:** penile cancer, epigenetic, DNA methylation, molecular markers

## Abstract

Penile carcinoma (PeCa) represents an important public health problem in poor and developing countries. Despite its unpredictable behavior and aggressive treatment, there have only been a few reports regarding its molecular data, especially epigenetic mechanisms. The functional diversity in different cell types is acquired by chromatin modifications, which are established by epigenetic regulatory mechanisms involving DNA methylation, histone acetylation, and miRNAs. Recent evidence indicates that the dysregulation in these processes can result in the development of several diseases, including cancer. Epigenetic alterations, such as the methylation of CpGs islands, may reveal candidates for the development of specific markers for cancer detection, diagnosis and prognosis. There are a few reports on the epigenetic alterations in PeCa, and most of these studies have only focused on alterations in specific genes in a limited number of cases. This review aims to provide an overview of the current knowledge of the epigenetic alterations in PeCa and the promising results in this field. The identification of epigenetically altered genes in PeCa is an important step in understanding the mechanisms involved in this unexplored disease.

## 1. Introduction

Penile carcinoma (PeCa), although relatively rare in developed countries of the world, is associated with high morbidity and mortality rates in poor and developing countries. The incidence of PeCa varies among populations, with an incidence rate of 0.58 and 0.84 per 100,000 males in United States and in the Western world, respectively [1,2]. In contrast, this rate is higher in developing countries, ranging from 2.3 to 8 cases per 100,000 men [3]. In Brazil, the incidence rate of penile cancer varies between 2.9 and 6.8 per 100,000 men and represents 2.1% of all cancer cases in men [4,5]. The mean age at presentation of penile cancer is 60 years [6].

The involvement of regional lymph nodes is the best indicator of long-term survival in patients with invasive penile carcinomas [7]. In addition to lymph node metastasis, other pathological factors, including grade, histological type, lymphovascular embolization, and stage and perineural invasion, have been described to be of prognostic value in PeCa [8–10]. However, none of them can effectively predict outcome.

There is a lack of information regarding the molecular genetic and epigenetic alterations and PeCa. Several studies in other cancer types have focused on epigenetic alterations, and there are promising data from clinical trials regarding the targeting of genes that regulate epigenetic events [11,12]. However, the published studies on PeCa are limited to the evaluation of CpG islands in specific genes.

## 2. Risk Factors: Human Papillomavirus as One of the Main Actors

Several risk factors have been associated with the development of malignant penile lesions. The most important risk factors are the presence of phimosis [13], poor genital hygiene [14], tobacco usage [15], and human papillomavirus (HPV) infection [16].

A history of phimosis is found in approximately 25% of penile cancer patients [2,17] and is strongly associated with invasive PeCa [17,18]. While circumcision shortly after birth is a protective factor, it does not have the same protective potential when carried out later in life [19]. The lack of circumcision, together with poor genital hygiene, contributes to the accumulation of smegma (which forms from desquamated epithelial cells) and consequently to the risk of developing PeCa [14]. The treatment of psoriasis patients with psoralen and ultraviolet A photo chemotherapy has also been identified as a risk factor for PeCa [20].

Human papillomavirus infection has been reported to have an important role in the development of a subset of PeCa, and its presence is thought to be related to the histological type [16]. Basaloid and warty penile cancers are very frequently HPV-positive, ranging from about 80% to 100%. Conversely, viral DNA is only found in a small fraction of verrucous penile carcinomas [20]. High-risk HPVs exert their oncogenic effect by expressing the oncoproteins, E6 and E7, which bind to and inactivate the tumor suppressor proteins, p53 and Rb, respectively [21].

It has been hypothesized that penile squamous cell carcinomas (PeSCC) arise by two distinct etiologic pathways. One is mediated by HPV infection and most likely involves sexual contact. The second occurs via a non-viral pathway and is related to other risk factors, such as poor genital hygiene and the presence of phimosis [22,23]. The incidence of HPV DNA found in penile carcinoma tissue ranges from 15% to 78% and varies according to the population studied, the method of specimen collection, and the protocol used for HPV detection [24].

In general, HPV infection, mainly by the HPV-16 and HPV-18 genotypes, is found in approximately 50% of all PeSCC [16,25] and is associated with multiple sexual partners [26]. Reports have indicated a positive association between the incidence of HPV infection in male sexual partners of women who had been diagnosed with cervical neoplasia [27]. Men vaccinated with the quadrivalent HPV vaccine that protects against HPV 6/11/16/18 have also been shown to have significantly less HPV-associated anogenital infection and PeCa [28].

Epstein-Barr virus (EBV) is another sexually transmitted virus that could be a cofactor in cancer development. The oncogenic potential of EBV is well known in lymphoproliferative disorders, such as Hodgkin’s lymphoma, Burkitt’s lymphoma, and lymphoma in immune compromised individuals [29]. The association between EBV and HPV has also been suggested in cervical cancer [30]. EBV is one of the most efficient cellular growth-transforming viruses known, and it has been found frequently in the genital mucosa, urethral discharges, and genital ulcers. In penile cancer, Epstein-Barr virus positivity was found in 46.7% of cases, and more than 23% of the men were co-infected with both HPV and EBV [31].

In addition, a number of other penile conditions have been associated with an increased risk of PeCa, including balanitis xerotica obliterans, Bowen’s disease, erythroplasia of Queyrat, bowenoid papulosis, and giant condyloma [32]. Bowen’s disease, erythroplasia of Queyrat, and bowenoid papulosis are uncommon pre-malignant disorders of the anogenital skin that may be confused with a variety of other lesions. Bowen’s disease has been reported to degenerate into invasive carcinoma in 5% to 10% of cases [33], while erythroplasia of Queyrat has been associated with invasive carcinoma in up to 30% of cases [34]. Penile lichen sclerosus, also known as balanitis xerotica obliterans, is a chronic inflammatory disorder that occurs in men of all ages [35], and which is associated with PeSCC in 4% to 6% of patients [36].

Overall, PeCa seems to be a multifactorial disease with several risk factors, including HPV and EBV infection, phimosis, poor hygiene habits, and tobacco usage. Several penile diseases have also been associated with a higher risk of developing PeCa.

## 3. Histopathological Data: Involvement of Lymph Nodes as the Major Predictive Factor of Poor Prognosis in Penile Cancer

Penile cancer usually originates in the epithelium of the inner prepuce (21% of cases), and the glans (48% of cases) [37]. Most penile malignancies (95%) are squamous cell carcinomas (SCCs). However, PeSCC represents a heterogeneous group of histopathological entities that differ in terms of morphology, pathogenesis, and prognosis [38]. The most common PeSSC type is the usual, followed by verrucous, papillary, basaloid, sarcomatoid, warty, cuniculatum, pseudohyperplastic, adenosquamous, and acantholytic [39]. The histological subtypes carry different risks of developing metastasis to lymph nodes, with usual SCC having a risk of 56.7% and sarcomatoid carcinoma having a risk of 89% [40].

PeSCC can also be divided into five categories according to its way of growth as follows: superficially spreading, vertical growth, verrucous, multicentric, and mixed [41]. Superficially spreading squamous cell cancers occur most frequently, and lymph node metastasis are present in 42% of cases. Lesions with a deeper vertical growth present with positive lymph nodes in 82% of cases. Multicentric lesions have positive lymph nodes in 33% of cases, whereas verrucous lesions rarely present with metastasis to the lymph nodes [42]. The pattern of growth in PeSCC and the depth of invasion are important prognostic determinants [10]. The histopathological grading is based on the Broder’s system that classifies the tumors in well-differentiated tumors (grade I) to undifferentiated and invasive tumors (grade IV) [43]. Two staging systems are used in penile carcinoma: the Jackson classification [44], and the TNM classification [45].

The presence of metastasis in regional lymph nodes is the main factor predicting an unfavorable prognosis for patients with PeSCC [9]. Palpable inguinal lymphadenopathy is present at diagnosis in 58% of patients (range from 20% to 96%), and metastatic carcinoma in 45% of these patients [37]. In the early stages of the disease, radical inguinal lymphadenectomy has been demonstrated to convey survival benefits [7,46]. However, these surgical techniques are limited by their high rates of morbidity and mortality [47]. Recent studies have shown new surgical techniques that have improved patient survival [48,49].

The prognosis of patients with lymph node metastasis varies according to the number of positive lymph nodes, the presence of uni- or bilateral inguinal extension, pelvic node involvement, and the presence of lymph node capsular involvement [9].

Kattan *et al.* [50] and Ficarra *et al.* [51] developed nomograms to predict inguinal lymph node involvement and the five-year cancer-specific survival of PeCa patients. These predictive models of patient outcome integrated the information about inguinal lymph node stage, pathologic tumor thickness, growth pattern, histologic grade, lymphatic and venous embolization, corpora cavernosa infiltration, corpus spongiosum, and urethral infiltration. Although nomograms allow improvements in prognostic accuracy compared with the use of each single variable, their use in clinical practice is potentially limited by the lack of external validation [9].

The studies evaluating the impact of HPV infection on the prognosis of patients with PeCa are controversial. Some studies have found an association between HPV positive infection and poor prognosis [52,53], while others have suggested that HPV status does not influence prognosis in invasive penile carcinoma [54–56]. HPV infection has also been related to favorable prognosis, as reported by Lont *et al.* [57], who showed a five-year cancer-specific survival rate of 92% for HPV-positive and 78% for HPV-negative patients. In the same study, the presence of positive lymph nodes was detected in 71% of HPV-negative cases, compared to 29% of HPV-positive patients.

## 4. Epigenetic Alterations and Cancer: Emerging Potential Markers of Diagnosis, Prognosis, and Therapy

Epigenetic modifications are potentially reversible alterations in DNA methylation or chromatin that are not associated with changes in the DNA sequence. These modifications specify functional outputs from the DNA template and are often heritable through cell division [58–61]. The epigenetic regulatory mechanisms are comprised of DNA methylation, histone modifications, and transcriptional alterations induced by noncoding RNAs. Aberrant epigenetic regulation can lead to alterations in global gene expression and genomic instability, which have been shown to have clear implications in the development of cancer [62].

DNA methylation changes include locus-targeted hypermethylation and global hypomethylation [63,64]. DNA methylation is catalyzed by a family of enzymes called DNA methyltransferases (DNMTs). These enzymes transfer a methyl group, donated by *S*-adenosylmethionine (SAM), to the fifth position carbon of cytosine. Three catalytically active DNMTs, DNMT1, DNMT3A, and DNMT3B are described in the mammals genome [65].

In mammals, DNA methylation primarily occurs by the covalent modification of cytosine residues in CpG dinucleotides. CpG dinucleotides are not evenly distributed across the human genome, but they are instead concentrated in short CpG-rich DNA stretches called “CpG islands” and in regions of large repetitive sequences [66–68].

CpG islands are preferentially located at the 5′ end of genes and occupy approximately 60% of human gene promoters [69]. While most of the CpG sites in the genome are methylated, the majority of CpG islands usually remain unmethylated during development and in differentiated tissues. However, some CpG island promoters become methylated during cancer development and progression. In contrast, the repetitive genomic sequences, retrotransposons, introns, and gene deserts, which are scattered throughout the human genome, become unmethylated during tumorigenesis. The global hypomethylation of these DNA regions during cancer development leads to increased genomic instability and results in chromosomal rearrangement [67,70].

The investigation of aberrant CpG island methylation has primarily been carried out using a candidate gene approach [71]. Several methods can be used to determine methylation patterns [67], including methylation-specific polymerase chain reaction (MSP) [72], MethyLight [73], combined bisulfite restriction analysis (COBRA) [74], methylation-specific single-nucleotide primer extension (MS-SNuPE) [75], methylation sensitive high resolution melting (MS-HRM) [76], and quantitative bisulfite pyrosequencing [77]. The gold standard technique to detect DNA methylation at a specific locus is quantitative bisulfite pyrosequencing, which analyzes bisulfite-modified and PCR-amplified DNA and provides information on the methylation status of individual CpG sites [77].

A large number of techniques are available for studying global DNA methylation. Genome-wide approaches can be broadly grouped into three strategies according to how DNA is modified before it is interrogated using microarrays or next generation sequencing platforms. These modifications include bisulfite converted DNA, affinity assays that precipitate methylated DNA (MeDIP and MCIP), and restriction enzyme methods that recognize methylated and unmethylated sequences (CHARM, LUMA, HELP) [78–83]. After the initial DNA enrichment or chemical modification, genome-wide analyses can be performed by array hybridization systems, such as the Illumina Infinium and GoldenGate systems (Illumina, Inc., San Diego, CA, USA), or oligonucleotide tiling arrays, such as the Nimblegen (Roche NimbleGen, Madison, WI, USA) and Agilent CpG Islands plus Promoters arrays (Agilent, Santa Clara, CA, USA), pyrosequencers, and next generation sequencing platforms such as Illumina/Solexa, ABI/SOLiD, Roche 454 and Helicos/Single molecule sequencing [84,85]. Although some of these techniques present biases or limitations, they are still useful for interrogating epigenetic marks, especially DNA methylation profiles.

Identifying changes in the methylation profile in tumors allows the identification of molecular markers for diagnosis and prognosis in cancer that could also be translated into therapeutic targets [77]. Wei *et al.* [86] reported that global hypomethylation was associated with worse prognosis or recurrence after treatment in ovarian cancer patients. In addition, methylation profiles have been demonstrated as important tools for the diagnosis of disease and the prediction of disease progression [86–89].

The cytosine methylation in CpG islands at promoter regions provides a stable gene silencing mechanism that plays an important role in regulating gene expression and chromatin architecture. Methylation often occurs in association with histone modifications and other chromatin-associated proteins. Histone proteins, which comprise the nucleosome core, contain a globular *C*-terminal domain and an unstructured *N*-terminal tail [90]. Post-translational modifications of histone tails determine which regions of the genome are in a transcriptionally active conformation or in a transcriptionally inactive form. The modifications of histone tails include acetylation, methylation, ubiquitylation, phosphorylation, sumoylation, and ribosylation. Each of these modifications regulate key cellular processes, such as transcription, replication, and repair [91–93].

Histone modifications can lead to either the activation or the repression of target genes, depending on the specific residues modified and the type of modifications present. Several active and repressive histone modifications have been identified, and these constitute a complex gene regulatory network in cells, which is known as the “histone code” [94]. The importance of epigenetic regulation is highlighted by the disruption of multiple epigenetic marks in various disease states. This is commonly associated with the deregulation of miRNA expression.

miRNAs are small, noncoding RNAs that regulate gene expression at the posttranscriptional level and are critical in many biological processes and cellular pathways [95,96]. miRNA expression profiles of human cancers have been described in several tumors, and the main causes of the aberrant miRNA expression patterns are DNA copy number alterations, the failure of miRNA post-transcriptional regulation, and genetic mutation or transcriptional silencing associated with the hypermethylation of CpG island promoters [96–101]. Recent studies have identified a number of miRNAs as potential biomarkers of diagnosis and prognosis, as well as targets for cancer therapy [102].

In recent years, remarkable progress has been made in target identification, drug discovery, and clinical validation for epigenetic therapeutics [103]. Inhibitors of two classes of epigenetic enzymes, *i.e.*, DNA methyltransferases (DNMTs) and histone deacetylases (HDACs), have already demonstrated utility as molecularly targeted chemotherapeutic agents for specific cancers and have received approval for these indications [11]. Furthermore, a database called HEMD (Human Epigenetic Enzyme & Modulator Database), which integrates human epigenetic enzymes and their modulators, has been developed to facilitate the investigation of epigenetic mechanisms and to provide subsidies for novel drug design [104].

Epigenetic alterations are present in all steps of cancer development and progression. With the improvement of techniques in the epigenetic field, especially those identifying global profiles, potential markers for diagnosis, prognosis and therapy have emerged for a series of tumors. Large-scale studies were also important in improving the knowledge about the mechanisms involved in several cancers. However, there is a lack of information regarding both genetic and epigenetic factors that are involved in PeCa.

## 5. Epigenetics Studies in PeCa

To the best of our knowledge, there are eight studies in the literature describing epigenetic alterations in PeCa [105–112], most of which evaluated the methylation pattern of CpG islands in specific genes (Table 1). Six of these studies investigated the CpG island status of *CDKN2A*. The *CDKN2A* locus encodes two tumor suppressor proteins, p16INK4A and p14ARF, which control cell growth through the Rb-CDK4 and p53 pathways, respectively [113]. The tumor suppressor gene *CDKN2A* blocks the cyclin-dependent kinases 4 and 6, which are involved in the activation of the cell cycle and the inhibition of CDK-mediated phosphorylation of the *RB* gene. Furthermore, the epigenetically mediated loss of *CDKN2A* is one of the most common and earliest events in human cancers [114].

**Figure 1:**
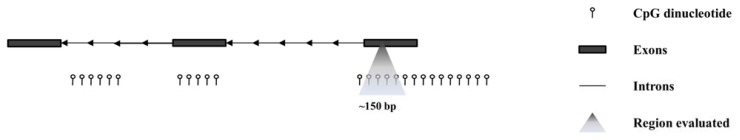
CpG islands described on *CDKN2A* gene. Three studies evaluated the same CpG island in an amplicon with 150 bases pair [106,108,112].

Considering all studies of methylation in PeCa, the promoter of *CDKN2A* has been globally investigated in 183 cases, and its methylation levels vary from 0% to 42% (Table 1). Reports with a larger number of individuals also presented a higher frequency of *CDKN2A* methylation [105,108,112]. Three studies evaluated the same CpG island and an amplicon with 150 bp [106,108,112] (Figure 1). Two of these studies found similar frequencies of methylation [108,112] (Table 1); however, Soufir *et al.* [106] reported 0% of methylation level. The low frequency detected by these authors may be related to the small number of invasive carcinomas studied (3 samples) or to HPV infection, which was found in two out of three PeCa samples. The *CDKN2A* primer sequences were not available in other studies [105,109,110].

Ferreux *et al.* [105] suggested at least three plausible mechanisms that could be involved in the disruption of the p16INK4A/cyclinD/Rb pathway during penile carcinogenesis, specifically, high-risk HPV infection, *CDKN2A* promoter methylation and *BMI-1* overexpression, which is an alternative mechanism that down-regulates *p16INK4A* [115]. A significant overexpression of BMI-1 was detected in tumors without methylation of the *CDKN2A* gene promoter. The data revealed that strong p16INK4A immunostaining was significantly associated with carcinomas positive for high-risk HPV. In addition, the frequency of *CDKN2A* promoter methylation was higher in HPV-negative tumors than in positive cases. According to Guerrero *et al.* [108], the hypermethylation of *CDKN2A* was correlated with negative and weak expression of the p16 protein, and all of the HPV-negative cases had weak or no p16 expression. The difference in p16 expression and the methylation patterns of *CDKN2A* between HPV positive and negative cases reinforce the hypothesis that PeCa is etiologically heterogeneous and may develop by distinct pathways. Furthermore, in other squamous cell carcinomas, there is evidence that differences in HPV status can lead to different tumor behavior and patient prognosis [116,117].

Poetsch *et al.* [112] investigated the effect of loss of heterozygosity (LOH), immunohistochemistry, point mutations and promoter methylation of *CDKN2A*. Fifty percent of primary PeCa showed p16INK4A overexpression, and cases that were negative for p16INK4A expression showed LOH near the *CDKN2A* locus and/or hypermethylation of the gene promoter. The absence of p16INK4A protein expression, LOH and promoter hypermethylation was significantly associated with the occurrence of lymph node metastasis. While p16 overexpression is almost always associated with the presence of HPV DNA in cervical carcinomas, Poetsch *et al.* [112] showed that the overexpression of p16INK4A, which is a frequent event in penile carcinomas, occurs in both HPV-positive and HPV-negative cases. The authors suggested that other pathways leading to the coactivation of p53 and p16INK4A that are independent of HPV must be considered because they found the expression of p16INK4A and p53 without the presence of HPV-DNA.

The reported methylation frequencies for *RASSF1A* varied from 11.5% to 45% (Table 1) [109,110]. Guerrero *et al.* [108] also investigated the expression of Thrombospondin-1 (TSP-1) and the methylation status of its promoter region. TSP-1 is a cell adhesion glycoprotein secreted by several types of normal cells and by tumor cells [118]. The hypermethylation of the *TSP1* gene was associated with unfavorable histological grade, vascular and tumor invasion, weak expression of TSP-1 protein and shorter overall survival. The association of the hypermethylation of *TSP1* with poor prognosis makes it a potential marker that could be used to detect more aggressive penile tumors. The methylation pattern of eight genes, *i.e.*, *DAPK*, *FHIT*, *MGMT*, *CDKN2A* (region p16INK4A and p14ARF), *RARβ*, *RASSF1A*, and *RUNX3*, revealed that at least one of them was methylated in each case [109]. In particular, the tumor suppressor gene, *FHIT*, and the gene, *RUNX3*, were methylated in 88% and 42% of the cases, respectively. Subsequently, the authors evaluated the same genes in 25 PeCa cases and included the FHIT protein expression by immunohistochemical staining [110]. Hypermethylation was detected in 92% of the cases, and decreased expression levels of FHIT protein were shown in 88% of cases. Twenty out of the 22 cases negative for FHIT protein expression showed *FHIT* methylation. Five genes, *DAPK*, *MGMT, CDKN2A*, *RARβ*, and *RUNX3*, were methylated in more than 20% of the cases. According to the authors, because the methylation of the *FHIT* gene was more common than the presence of HPV infection, which occurred in less than 5% of patients, this gene might play an important role in the pathogenesis of penile squamous cell carcinoma.

**Figure 2:**
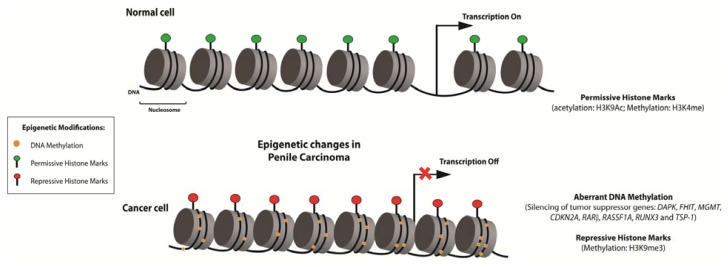
Epigenetic alterations were described in eight studies of penile carcinoma (PeCa). Aberrant DNA methylation pattern of *DAPK*, *FHIT*, *MGMT*, *CDKN2A*, *RARβ*, *RASSF1A*, *TSP-1*, and *RUNX3* and alteration in the expression levels of histones were events related with this disease. In special, *CDKN2A* gene was evaluated in six studies.

Recently, Rogenhofer *et al.* [111] evaluated the global methylation levels of the histones, H3K4, H3K9 and H3K27, on a tissue microarray platform containing 65 penile carcinomas, six metastatic lesions, and 30 normal skin samples by immunohistochemistry. A variation in the overall level of histone methylation was detected between normal and tumor samples. The overall levels of H3K4me1, H3K9me1, H3K9me2, H3K27me2, and H3K27me3 were decreased, whereas H3K9me3 levels were increased in PeCa. Hierarchical clustering analysis demonstrated that cancer and normal tissues were differentiated based on the histone methylation pattern of H3K9 and H3K27. A trend towards increased global histone methylation levels was detected in metastasis, and high H3K9me2 levels could be related to poor outcomes in PeCa patients. The epigenetic alterations described in PeCa are summarized in Figure 2.

At present, only a single study has investigated the pattern of methylation of genes in the HPV virus of men with PeCa. According to Kalantari *et al.* [107] the mechanisms involved in penile carcinogenesis related to HPV infection are similar to those involved in cervical carcinoma. The authors investigated three properties of the HPV genomes in penile carcinomas patients, specifically, the methylation of HPV DNA, the junctions between HPV and cellular DNA, and the genomic variation. The authors found that the HPV16 and 18 *L1* genes showed similar patterns of hypermethylation in penile and cervical carcinomas, and as such, the methylation of HPV16 and 18 *L1* DNA can serve as a biomarker of integration between HPV and cellular DNA in PeCa.

In summary, a substantial variability of methylation has been described for *CDKN2A* and *RASSF1A* in PeCa. Additionally, gene silencing through CpG island hypermethylation and FHIT downregulation have been suggested as potential markers in PeCa. Although several genes have been described to be epigenetically regulated in PeCa, the available data are limited, and only a few reports have confirmed the analysis using gene or protein expression.

## 6. Future Perspectives and Direction

It has been established that epigenetic changes are critical for the development and progression of several tumors. The majority of studies regarding epigenetic alterations in PeCa have only evaluated the patterns of specific genes. However, the assessment of relevant markers for this disease requires methods that detect alterations in methylation on a genome-wide level. Large-scale studies in PeCa are needed to better comprehend tumor behavior and to determine the molecular markers involved in this disease. In a recent review in penile cancer, Sonpavde *et al.* [119] emphasized the importance of a better understanding of the basic biology of PeCa to guide the design of clinical trials. In our opinion, given the significant number of PeCa cases that are positive for HPV and EBV infections, it is a necessity to investigate epigenetic alterations based on these patterns using more robust, genome-wide methods. These studies may identify new molecular markers that could be useful for designing effective therapeutic strategies against this clinically and psychologically aggressive disease.

## Figures and Tables

**Table 1:** Summary of the epigenetics studies in penile carcinomas described in the literature.

References	Number of samples	Method	Gene studied	% methylation	HPV infection	HPV 16
Ferreux *et al.* [105]	53	Methylation-specific PCR	*CDKN2A*	9 (17%)	20 (38%)	15 (28%)
Poetsch *et al.* [112]	52	Methylation-specific PCR	*CDKN2A*	22 (42%)	20 (38%)	18 (35%)
Soufir *et al.* [106]	3	Methylation-specific PCR	*CDKN2A*	0 (0%)	2 (66.3%)	2 (100%)
Guerreto *et al.* [108]	24	Methylation-specific PCR	*CDKN2A* *RASSF1A* *TSP-1*	9 (38%) 10 (42%) 11 (46%)	11 (46%)	10 (42%)
Yanagawa *et al.* [109]	26	Methylation-specific PCR	*DAPK* *FHIT* *MGMT* *CDKN2A (p16INK4A)* *CDKN2A (p14ARF)* *RARβ* *RASSF1A* *RUNX3*	7 (26.9%) 23 *(*88.4%) 5 (19.2%) 1 (3.8%) 6 (23.1%) 6 (23.1%) 3 (11.5%) 11 (42.3%)	3 (11.5%)	3 (11.5%)
Yanagawa *et al.* [110]	25	Methylation-specific PCR	*DAPK* *FHIT* *MGMT* *CDKN2A (p16INK4A)* *CDKN2A (p14ARF)* *RARβ* *RASSF1A* *RUNX3*	7 (28%) 23 (92%) 5(20%) 1 (4%) 6 (24%) 6 (24%) 3 (12%) 11 (44%)	3 (12%)	3 (12%)
Kalantari *et al.* [107]	24	DNA sequencing	L1 HPV16 LCR HPV16	58% 22%	24 (100%)	19 (79%)
Rogenhofer *et al.* [111]	65	Immunohistochemical	H3K4 H3K9 H3K27	H3K4me1 Decrease H3K9me1 Decrease H3K9me2 Decrease H3K27me2 Decrease H3K27me3 Decrease H3K9me3 Increase		
